# Household structures and exposure networks among people with multidrug-resistant TB in Namibia

**DOI:** 10.5588/ijtldopen.26.0039

**Published:** 2026-09-15

**Authors:** E. Iipumbu, G. Hoddinott, J.A Seddon, O. Shavuka, L. Boois, N. Shinedima, V. Haimbala, L. Absalom, L. Shiweva, H. Ekandjo, S. Sibela, M. Claassens

**Affiliations:** 1 Department of Human, Biological and Translational Medical Sciences, School of Medicine University of Namibia, Windhoek, Namibia;; 2 School of Public Health, Faculty of Medicine and Health, The University of Sydney, Sydney, NSW, Australia;; 3 Desmond Tutu TB Centre, Department of Pediatrics and Child Health, Stellenbosch University, Cape Town, South Africa;; 4 The University of Sydney Infectious Diseases Institute (Sydney ID), Sydney, NSW, Australia;; 5 Department of Infectious Disease, Imperial College London, London, UK.

**Keywords:** tuberculosis, kinship mapping, qualitative research, southern Africa, co-residence, mobility

## Abstract

**BACKGROUND:**

Multidrug-resistant TB (MDR-TB) poses major challenges for TB control in high-TB-burden settings such as Namibia. In southern Africa, family and household structures are fluid, yet little is known about how MDR-TB interacts with these structures. We therefore explored family structures, household changes, and implications for transmission and care among people living with MDR-TB in Namibia.

**METHODS:**

We conducted a qualitative sub-study within the Hotspots, Hospitals and Households (H3TB) transmission of TB project. Between June 2021 and August 2023, 119 participants with MDR-TB were recruited from three regions of Namibia. In-depth interviews using participatory kinship, social network, and community mapping tools were conducted. Data were thematically analysed, with case studies illustrating household reconfigurations.

**RESULTS:**

We identified 11 household structure patterns. Fourteen participants reported changes in household composition following MDR-TB diagnosis, driven by moving closer to health services, reorganising to optimise family care, reducing perceived exposure risks, and mitigating financial strain. These reconfigurations altered co-residence patterns and potential TB contact networks, with implications for household-based active case finding.

**CONCLUSION:**

MDR-TB acts as a stressor prompting household reorganisation, reshaping networks of exposure, care, and support. Accounting for dynamic household contexts may strengthen TB contact tracing and patient support strategies.

TB, caused by the bacterium *Mycobacterium tuberculosis* (*Mtb*), is a public health priority, with an estimated 10 million people developing the disease and 1.6 million deaths annually worldwide.^1^ Approximately 25% of the world's population has been infected with *Mtb*, and 5%–10% of people infected will develop TB disease during their lifetime.^2^ Approximately half a million of the individuals who develop TB each year have multidrug-resistant TB (MDR-TB, defined as TB caused by *Mtb* strains resistant to at least isoniazid and rifampicin, the two most effective first-line anti-TB drugs.^3^ Namibia is one of the WHO high-TB-burden countries, with an estimated incidence of 460 per 100,000 population and around 800 new MDR-TB cases annually.^1^ TB, including MDR-TB, is transmitted via inhalation of droplets expelled by a person with TB disease. Transmission risk is primarily influenced by exposure-related factors including duration and intensity of contact, and the infectiousness of the index patient.^4^ A whole-genome sequencing study in Namibia demonstrated geospatial clustering of MDR-TB transmission, likely linked to contact networks.^5^

Understanding the family and social networks of MDR-TB patients may help identify transmission chains and inform interventions such as active case finding, timely testing, preventive therapy, and treatment.^6,7^ Engaging with family and social networks may also improve treatment support, reduce stigma, and promote preventive measures within the community.^8^ In southern Africa, family networks are typically complex and fluid, extending beyond the conventional nuclear households to include extended kin and socially connected non-relatives.^9^ High individual and population mobility further shapes household composition, as people move between homes for economic, caregiving, cultural, and social reasons.^10^ Research from neighbouring countries (South Africa and Zambia) has shown that family structures influence health outcomes and access to care.^9^ However, little is known about how these family structures interact with TB, particularly MDR-TB.

In this study, we aimed to describe the family structure-associated exposure patterns among people living with MDR-TB in Namibia. Specifically, we 1) describe the types of family structures we observed and 2) use case examples to illustrate how MDR-TB disease episodes interface with family structure, perceived exposure risks, and care needs.

## METHODS

The Hotspots, Hospitals and Households (H3TB) transmission of TB project uses whole-genome sequencing, social, and geographic mapping to identify transmission ‘hotspots’ and evaluate enhanced case-finding strategies.^11^ As part of the H3TB project, we collected qualitative data on household contacts, social networks, and people’s use of community spaces.

### Setting

Namibia covers an area of approximately 825,000 km^2^. The study was conducted in three regions (counties/provinces): Khomas, centrally located (population 494,604); Ohangwena, northern part of Namibia (population 337,729); and Otjozondjupa located in the northeastern-central (population 220,811).

### Sampling and recruitment

Eligible participants were individuals diagnosed with MDR/RR-TB in the three regions between June 2021 and August 2023. Participants were purposively sampled to ensure diversity in age, gender, and geographic location, and recruitment continued until data saturation was reached.^12^ A research nurse linked to the national drug-susceptibility testing laboratory identified potential participants. Graduate researchers contacted participants telephonically or in person at the hospital. In rural/remote areas, community-based health care workers, from the National TB and Leprosy Programme, assisted with participant tracing.

### Data collection

For this study, ‘family’ encompassed all individuals identified by the participant as part of their kinship or close relational network, regardless of co-residence. A ‘household’ referred to individuals sharing living arrangements and domestic resources, while ‘co-residence’ described individuals sharing a dwelling at a given time, regardless of familial relationship.

Trained graduate researchers (EI, OS, LB, LA, SS, VH, and NS) conducted semi-structured in-depth interviews. Interviews included three sub-sections/participatory activities: 1) creating a kinship map using standardised symbols as seen in the key below each figure, 2) mapping the participants’ social networks, and 3) creating a geographic map of the participants’ communities. Kinship maps visually represented family relationships and co-residence patterns using standardised symbols.^9^ Interviews were conducted in a private hospital space, lasted ∼90 min, and were conducted in participants’ preferred languages, primarily English, Oshiwambo, Afrikaans, Portuguese, or Khoekhoegowab to ensure comfort and clarity. All interview notes, transcripts, and participatory outputs were subsequently prepared in English. Detailed field notes were expanded during weekly debriefing meetings (between the data collection team and GH) and retained for analysis.^13^

### Data analysis

The data collection team supervised by GH held monthly debriefing meetings to iteratively refine our understanding of the data throughout. Data were analysed thematically, using an inductive approach.^14,15^ Interview transcripts, field notes, and outputs from participatory activities were reviewed and coded to identify patterns in family structure and co-residence. Initial codes were developed from the data and iteratively refined into broader themes through repeated reading and comparison across cases – including through fortnightly analysis-focused meetings between EI and GH. Household structure patterns were developed through an iterative process of grouping similar configurations of kinship and co-residence identified across participants. These patterns were compared, discussed, and refined within the research team to ensure consistency and interpretive rigour. Case-study analyses examined changes in household structure and how these interfaced with MDR-TB diagnosis and care.^16,17^

### Ethical statement

Ethics approval for the H3TB project including this analysis was obtained from the University of Namibia Research Ethics Committee (UREC) [HGC/611/2021] and the Ministry of Health and Social Services (MOHSS) Research Office. Written informed consent was obtained from all participants; for participants under 18 years, parental consent and child assent were obtained.

## RESULTS

We identified 119 eligible participants balanced by gender and with an age distribution reflecting TB epidemiology in Namibia as seen in Table 1. Sixty-seven were from the Otjozondjupa region, 26 from the Ohangwena region, and 26 from the Khomas region. Twenty self-reported that they were living with HIV.

**Table 1.** Participant demographics and characteristics of individuals recruited to the study.

	Total	Region		
		Khomas	Otjozondjupa	Ohangwena
Age group				
≤18 years	16	2	13	1
19–39 years	66	16	36	14
40–59 years	28	7	14	7
60–79 years	5	1	2	2
≥80 years	1	0	0	1
Unknown	3	0	2	1
Gender				
Male	60	13	31	16
Female	59	13	35	11

### Family structures

**Figure 1.**
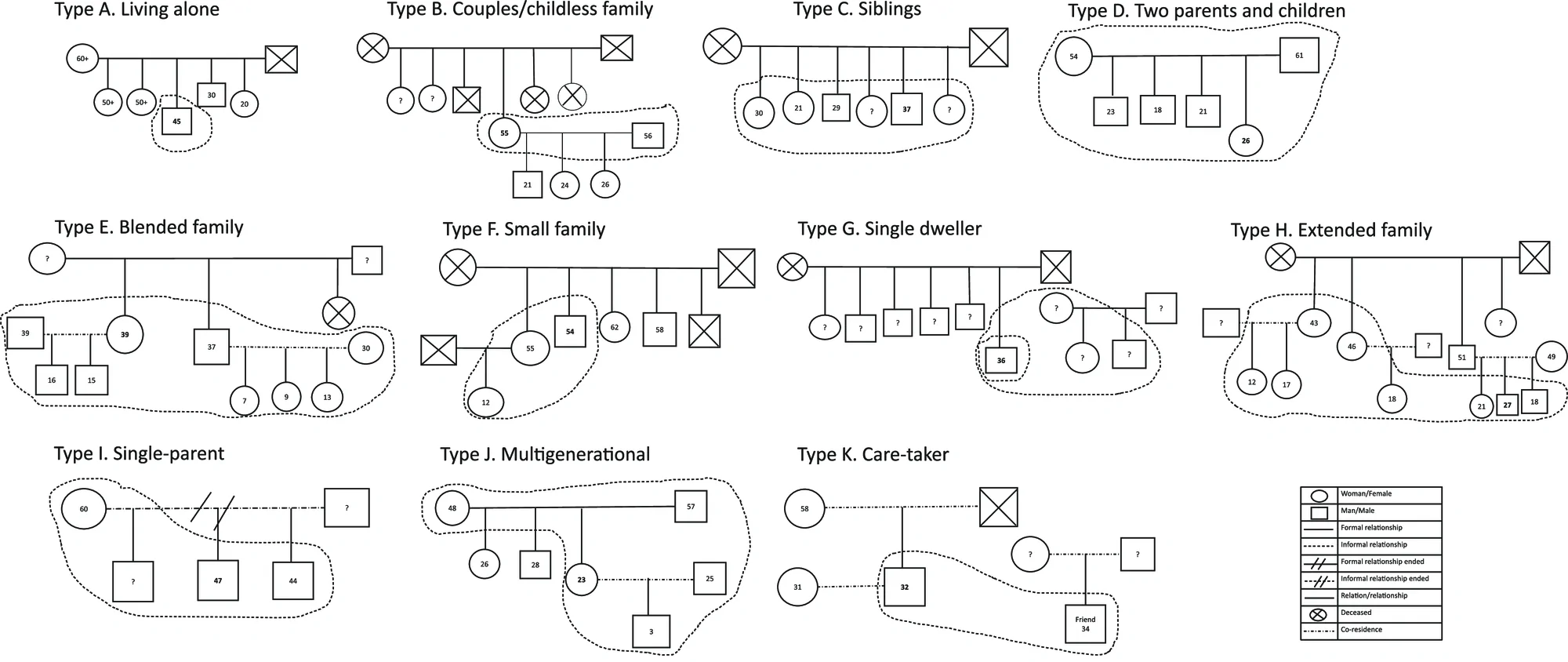
Types of household structures identified.

We identified 11 patterns of household structure and co-residence patterns (Figure 1). For example, participant A was a 29-year-old man who lives alone in Okahandja. Both his parents and his three sisters are alive, but he was not co-resident with any of them. He is not currently in a relationship, and he does not have children. This participant was grouped into the Type A ‘living alone’ pattern. In contrast, participant G was a 55-year-old man who lives with his partner, a woman in her 40s. They are married, have two children together, and the four of them are co-resident. This man also has a second household where he lives with his other partner (intermittently), with whom he has an informal relationship. This woman has two children (participant is not the father), a 20-year-old daughter and an 18-year-old son with whom she is co-resident. This participant was grouped into both a Type D ‘Two parents and children’ pattern with his wife and a Type E ‘Blended family’ pattern with his girlfriend and her children. Overall, the most common pattern type was Type D ‘Two parents and children’ across all regions (Table 2). In Khomas, the second most common structure seen was Type F ‘small family’. In Ohangwena and Otjozondjupa, Types E ‘Blended family’ and J ‘Multigenerational household’ were also common.

**Table 2.** Household structure and co-residence patterns per region (n = 119).

Household structure	Region			Total
	Khomas	Otjozondjupa	Ohangwena	
Living alone	3	3	3	9
Couples	0	3	0	3
Two parents and children	7	30	4	41
Multigenerational	3	13	10	26
Extended family	0	1	0	1
Blended family	1	11	7	19
Single-parent family	4	1	0	5
Caretaker	1	0	0	1
Single dweller	0	1	0	1
Siblings	0	1	0	1
Small family	7	3	2	12

### Interface between MDR-TB and household structures

**Figure 2.**
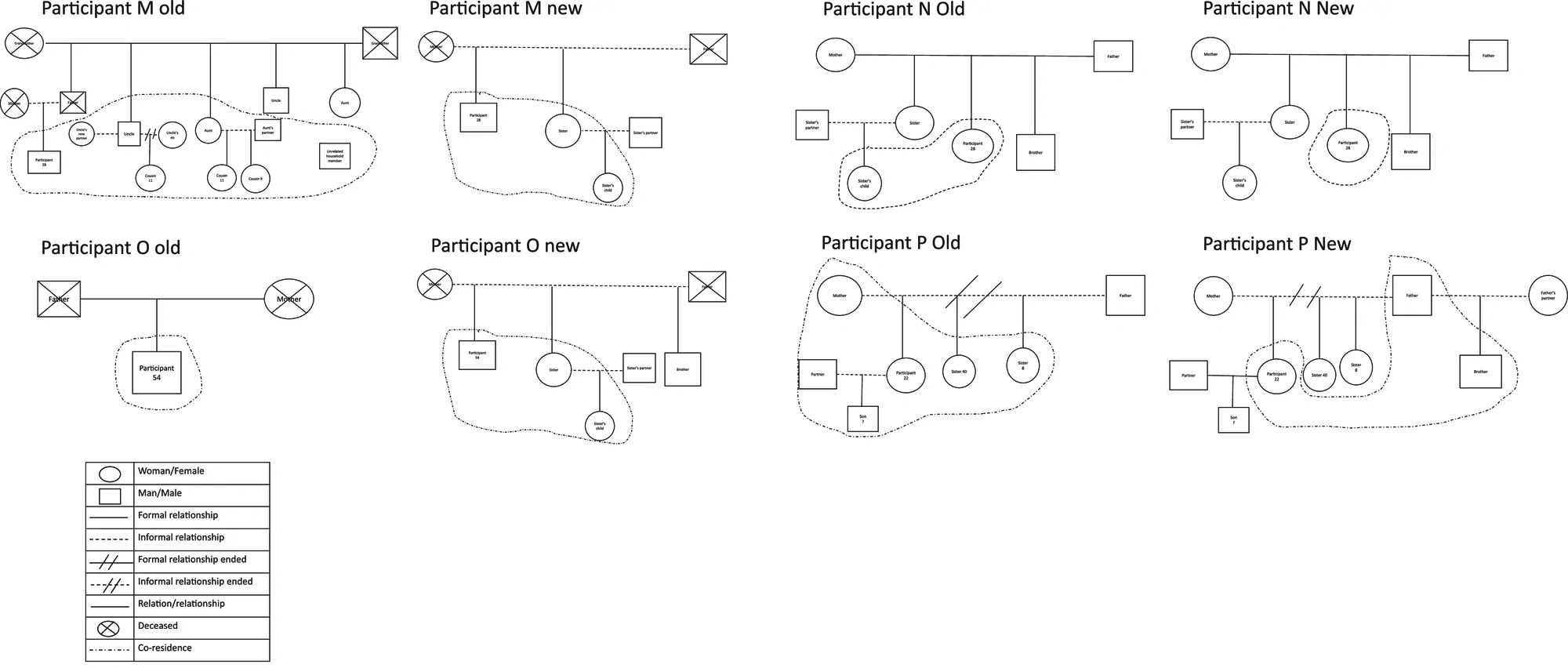
Changes in household structures.

We found that 14 of 119 families changed their structure in response to the MDR-TB disease episode. Even among participants who did not report changes to their family structures, many had considered doing so but either decided this was not feasible or opted not to do so having considered trade-offs. We identified four pathways by which finding out that they were living with MDR-TB was a stressor to their family and co-residence pattern. We present each in turn with illustrative examples (see Figure 2):

1. Moving closer to a health care service centre: participant M was a 28-year-old man who started his TB treatment in Angola. When he went back to the hospital after a few months to collect his medication in Angola, he was told that the hospital had run out of the required drugs and that he should purchase these from the pharmacy. He did not have the money to do so. His sister then invited him to move into her home (in Namibia) because she lives close to a hospital with an adequate stock of drugs. Participant M shifts from a Type E ‘Blended family’ to a Type F ‘Small family’. In the new home, he is co-resident with his sister and her daughter who are new ‘contacts’ but who would not have been identified as such since he considered his household as the extended family in Angola, not the ‘temporary’ one in his sister’s house.
2. Optimising familial care offered to the patient: participant O was a 54-year-old man who was living alone in the Oshana region. After he was diagnosed with MDR-TB, his sister (a medical doctor) realised that he was too ill to take care of himself. She invited him to move to Windhoek (in Khomas) and live with her and her daughter. Participant O shifted from a Type A ‘Living alone’ to a Type F ‘Small family’. Again, new co-residents are now potential MDR-TB contacts.
3. Re-structuring to avoid perceived exposure risk: participant N was a 28-year-old woman, who was living with her 8-year-old niece in the Khomas region. When participant N learned that she had MDR-TB, her niece was sent back to her parents in the Erongo region. Participant N shifted from a Type H ‘Caretaker’ to a Type A ‘Living alone’. The logic for this change was both because participant N and her sister and brother-in-law (the niece’s parents) were worried about risk of transmission to the niece, and because participant N felt unable to keep up with the physical and emotional demands of caring for an 8-year-old child.
4. Cost-saving to absorb the additional costs associated with the patient’s costs of care and/or loss of income: participant P was a 22-year-old woman who lived with her mother, her son (6-years-old), and two of her sisters (40- and 8-years-old, respectively) in a suburb of Windhoek in the Khomas region. In her household, she was the only employed person, and due to her illness, she could no longer work. She said: ‘When I got diagnosed with MDR-TB, the family I was living with moved out of Windhoek and went to the north. I then went to stay with my father and my 25-year-old brother in another part of Windhoek’. Participant P shifted from a Type J ‘Multigenerational’ to a Type I ‘Single-parent’ (in which her father is the parent). In her new circumstances, she was both financially dependent on her father and brother and unable to support her ‘family’ (son, mother, sister) as she had previously. She described this as a deeply distressing experience, as she could no longer provide for her mother, young son, and ill sister, whom she had been supporting with the little income she earned before her MDR-TB diagnosis.

We found that of the 14 participants who changed household and co-residence patterns because of TB, 4 were in response to moving closer to health care, 3 were to optimise familial care/support, 3 were to avoid perceived exposure risk, and 1 was cost-saving to absorb patient care costs/loss of income. Three did not want to specify why they moved.

## DISCUSSION

We identified 11 patterns of kinship and co-residence among people with MDR-TB in Namibia. For some families, MDR-TB prompted reorganisation of household structure to access health services, support care, reduce perceived exposure risk, or mitigate financial strain. These shifts influence contact networks with direct implications for household-based active case-finding strategies.

Our findings have important implications for MDR-TB case detection and contact management. Conventional contact tracing is largely centred on the household as a fixed unit; however, our findings show that household structures are fluid and frequently reconfigured following MDR-TB diagnosis. As a result, individuals at risk of exposure are not limited to those residing in the household at diagnosis. Expanding contact investigation to include broader social and co-residence networks, as well as recent and anticipated household movements, may improve case detection. However, such approaches present challenges, including increased resource requirements, difficulties tracking mobile populations, and the need for adaptable programmatic tools. Our findings are consistent with studies from South Africa and Zambia among households affected by HIV,^9^ and with broader descriptions of household structures in southern Africa.^18,19^ To our knowledge, this is the first study describing these patterns among families affected by MDR-TB and in Namibia. Previous qualitative research has described challenges faced by people with TB and MDR-TB in accessing care and navigating family and social support.^20–23^ Our study extends this by showing how MDR-TB acts as a stressor that reshapes household structure and co-residence patterns. Similarly, previous research has highlighted challenges in active case finding in settings such as prisons, mines, informal settlements, and rural areas.^24–27^ Mobility and under-reporting of contacts remain key barriers.^28^ Our findings begin to chart an approach to eliciting broader contact networks among TB patients.

A key strength of this study is that it is the first to systematically explore household structures and co-residence patterns among families affected by MDR-TB in Namibia. By focussing on this often-overlooked dimension, the study provides novel insights into how MDR-TB can prompt reorganisation of family living arrangements. Integration within the larger Hotspots, Hospitals and Households project, and collaboration with the MOHSS, enhances the potential for translation of findings into policy and practice. The use of participatory tools such as kinship mapping and household diagrams enabled detailed exploration of how family configurations influence, and are influenced by, MDR-TB disease episodes. These methods allowed identification of household changes related to access to care, caregiving, perceived exposure risk, and financial strain. These findings offer important considerations for refining household-based contact tracing and care strategies in TB-control programs.

Limitations include transferability, which is limited by the study’s focus on three Namibian regions, as household structure is context-dependent and findings may differ elsewhere. Additionally, recruitment through the national drug-susceptibility testing laboratory excluded undiagnosed individuals who may have poorer health care access and different household structures potentially limiting the representativeness of findings.

Our findings underscore that household contacts are not static: individuals living with a patient before diagnosis may differ from those present after diagnosis. Accounting for these shifts could improve contact tracing and risk assessment. While this study did not quantify contacts before and after household reconfiguration, such analyses could illuminate differences between conventional household-based and expanded network-based approaches, informing resource allocation in TB-control programmes. Future studies should explore these dynamics using quantitative methods to better characterise contact patterns and guide targeted TB-control measures.

## CONCLUSION

Households affected by MDR-TB are dynamic, and MDR-TB itself acts as a stressor that reshapes co-residence and networks of exposure, care, and support. Contact tracing, case finding, and patient support strategies in high-burden settings must be agile to adequately serve those affected.
